# Different labour outcomes in primiparous women that have been subjected to childhood sexual abuse or rape in adulthood: a case–control study in a clinical cohort

**DOI:** 10.1111/1471-0528.12053

**Published:** 2012-11-12

**Authors:** H Nerum, L Halvorsen, B Straume, T Sørlie, P Øian

**Affiliations:** a Department of Obstetrics and Gynaecology, University Hospital of North NorwayTromsø, Norway; b Department of Clinical Medicine, University of TromsøTromsø, Norway; c Department of Community Medicine, University of TromsøTromsø, Norway; d Department of General Psychiatry, University Hospital of North NorwayTromsø, Norway

**Keywords:** Birth outcomes, caesarean section, childhood sexual abuse, duration of labour, rape

## Abstract

**Objective:**

To compare the duration and outcome of the first labour in women who have been subjected to childhood sexual abuse (CSA) and women who have been raped in adulthood (RA).

**Design:**

Case–control study in a clinical cohort.

**Setting:**

University Hospital of North Norway.

**Sample:**

In all, 373 primiparas: 185 subjected to CSA, 47 to RA and 141 controls without a history of abuse.

**Methods:**

Data on birth outcomes were retrieved from the patient files. Information on sexual abuse was reported in consultation with specialised midwives in the mental health team. Birth outcomes were analysed by multinominal regression analysis.

**Main outcome measures:**

Vaginal births, delivery by caesarean section, operative vaginal delivery and duration of labour.

**Results:**

As compared with controls, the RA group showed a significantly higher risk for caesarean section (adjusted OR 9.9, 95% CI 3.4–29.4) and operative vaginal delivery (adjusted OR 12.2, 95% CI 4.4–33.7). There were no significant differences between the CSA and the control group. The RA group displayed significantly longer duration of labour in all phases as compared with the control and CSA groups.

**Conclusions:**

There were major differences in the duration of labour and birth outcomes in the two abuse groups. Despite a higher proportion of obstetric risk factors at onset of labour in the CSA group, women subjected to CSA had shorter labours and less risk for caesarean section and operative vaginal deliveries than women subjected to RA. The best care for birthing women subjected to sexual abuse needs to be explored in further studies.

## Introduction

Sexual abuse leads to extensive injurious health effects. Several studies have shown that childhood sexual abuse (CSA) and rape in adulthood (RA) lead to problems such as sexual dysfunction, anxiety, depression, post-traumatic stress disorder, eating and sleep disturbances, functional gastrointestinal disorders and chronic pain, such as headaches, fibromyalgia and pelvic pain.^1^–^7^ It has been shown that risk-taking behaviour and self-destructive behaviour such as smoking, substance abuse and suicide attempts are strongly associated with CSA.^1^,^2^,^8^ Similar behaviour is reported by women subjected to violence, sexualized violence and rape in adulthood.^2^,^5^,^9^–^11^ Women with a history of CSA or RA often seek help from the health services for various somatic symptoms without connecting these symptoms to the history of abuse.^1^,^5^,^7^,^12^,^13^ In others, negative feelings such as shame and guilt may prevent them from contacting the health services.

The reported prevalence for sexual abuse varies depending on the different samples, research settings, methodology, definitions used and when in life the abuse occurred. Estimates indicate that 6–36% of women have been subjected to forced sexual activity or sexual violence.^3^,^7^,^9^,^12^–^17^ Among pregnant women, 7–37% have reported CSA.^18^,^19^,^22^ Our research group has reported that 63% of pregnant women with moderate and serious fear of birth have been subjected to physical, emotional or sexual abuse.^20^ Women with a history of CSA report more physical and emotional health problems in pregnancy, such as stress, anxiety, depression and suicidal thoughts, than women without such experiences.^8^,^16^,^21^,^22^ A strong association between CSA and post-traumatic stress symptoms in pregnancy has been reported.^8^,^18^

The few studies that have examined a possible association between sexual assault and birth outcome show equivocal results. In most studies no increased risk of complications in labour is observed in women who have been subjected to CSA.^16^,^18^,^23^ It is difficult to compare these studies because the methods used for charting the abuse experiences vary, samples are different and little distinction is made between various forms of abuse, when in life the abuse has occurred or parity. Lukasse et al. showed that primiparas exposed to any kind of physical, emotional and sexual abuse in childhood had a slightly increased risk for caesarean section during labour.^24^

In a recent study, we showed that women who had previously been subjected to RA had a longer second stage of labour and an increased risk of caesarean section and operative vaginal delivery, compared with controls from the general birth cohort.^25^ In our clinical practice we had the impression that it was easier for women who had been subjected to CSA to give birth vaginally than for women who had been subjected to RA or who had no history of abuse. This led us to hypothesise that CSA and RA groups may have different courses and outcomes in their first births.

The aim of this study was to compare the duration and outcome of labour in nulliparous women who had been subjected to either CSA or RA against a control group with no history of sexual abuse.

## Material and methods

**Figure 1:**
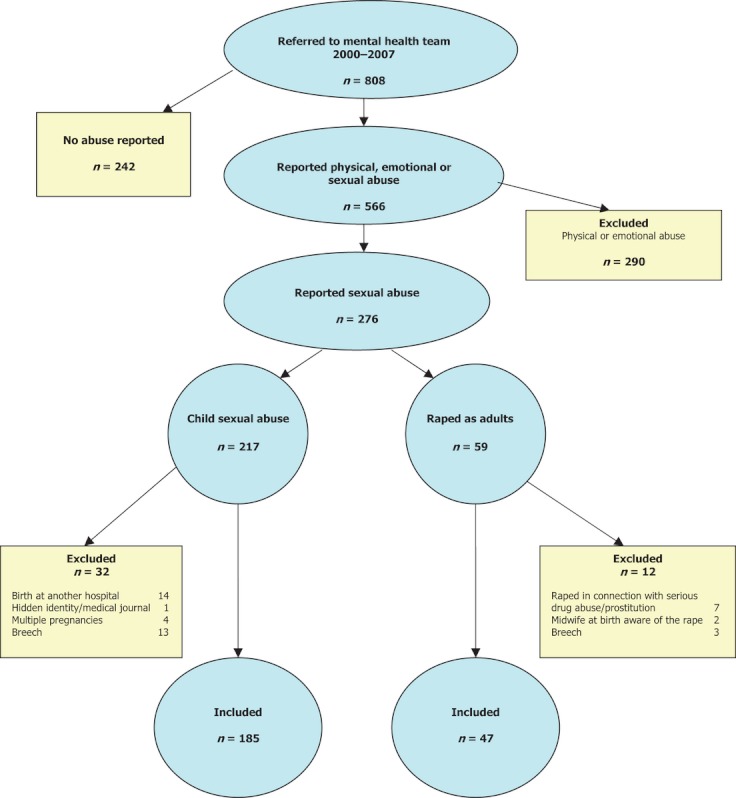
Flow chart of inclusion in the study.

The two abuse groups were recruited from a cohort of 808 parous women referred for care to the mental health team at the antenatal outpatient clinic, University Hospital of North Norway, in the period 2000–2007 (Figure 1). The women were referred in subsequent pregnancies for various psychosocial burdens, such as history of anxiety and depression, eating or sleep disturbances, post-traumatic stress disorder, fear of birth or a previous traumatic birth experience. Previous sexual abuse was unknown for the birth attendants at the first birth. The referred women received care with a crisis-oriented approach, as has been described previously.^20^,^26^ Abuse was systematically charted in the consultations according to what kind of abuse or assault it was, the woman's age at the time, who the perpetrator was, as well as where and how it occurred.

Inclusion criteria for the CSA group were having given birth to the first child at the University Hospital and having been subject to sexual acts or contact, which included touching the genitals, masturbation, oral sex, attempts at penetration of the vagina or sexual intercourse under the age of 16 years (the minimum age of sexual consent in Norway). Many of the abuse experiences were accompanied by physical violence and/or emotional threats. The abuse was perpetrated by parents, step-parents, grandparents, siblings, uncles, aunts, cousins, other caregivers, neighbours, teachers or leaders in voluntary organisations and religious congregations. Inclusion in the RA group was having been raped vaginally after the age of 16 years without any history of CSA.^25^ None of the women in this study gave birth to their first child as a result of sexual abuse. The control group was recruited from the same population of nulliparous women at the hospital, and was matched for age and year of birth against those who had been raped in adulthood. The CSA group was younger when giving birth for the first time, which is characteristic for abuse in childhood.^27^ We were unable to find matched controls without abuse for the youngest women in our general birth cohort. The exclusion criteria used are described in Figure 1.

### Variables

The following variables were collected from the electronic journal system Partus®: age, marital status, education, employment status, smoking habits, pre-pregnancy body mass index (BMI), miscarriages, pregnancy terminations and prenatal visits. Obstetric risk was defined as: having a chronic somatic illness of significance to the pregnancy or complications, such as diabetes, pre-eclampsia, polyhydramnion, oligohydramnion, intrauterine growth restriction, a gestation lasting more than 42 weeks, prelabour rupture of membranes without labour after 24 hours and meconium staining in the fluid. Spontaneous or induced labour was also registered: oxytocin augmentation, epidural, operative vaginal delivery, caesarean section, episiotomy and blood loss during birth.

The active part of first stage of labour was defined as starting at 4–cm dilation and lasting until dilation was complete. The latent phase of the second stage was defined as the period from complete dilation until active pushing, and the active phase of the second stage was defined as the time from the start of active pushing until the child was born. Fetal station in pelvis was registered at the start of active labour, at the latent and active phase of the second stage. The baby's birthweight, 5–minute Apgar score and transferrals to the neonatal intensive care unit within the first 4 days of birth were also registered.

For operative deliveries with combined indications the prime indication is registered. For acute caesarean sections the indication ‘failure to progress’ includes cephalopelvic disproportion, ineffective contractions, deflection and failed ventouse or forceps delivery. The indication ‘fetal distress’ includes pathological findings by auscultation or in combination with cardiotocography (CTG) or ST analysis (STAN) of the fetal electrocardiogram intrapartum.

### Statistical analysis

When comparing groups (CSA, RA and CG) the chi-square tests or Kruskal–Wallis tests were applied, as indicated. When computing odds ratios the CSA/RA groups were coded (1/0) as dummy variables, such that OR < 1 indicates a favourable outcome compared with the control group. To evaluate the risk of caesarean section or operative vaginal delivery, a multinomial logistic regression analysis was performed with the following three steps. Model 1, with mode of delivery as a dependent variable, and dummy variables for CSA and RA as independent variables, where all putative confounding factors were added ‘univariately’, was developed first. Variables that were significantly associated with either of the birth outcomes were then included in multivariable models 2 and 3, as presented in Table 1, and included variables that retained a significant relation to either operative vaginal delivery or caesarean section in model 1. *P* < 0.05 was the level of inclusion in all steps. Risks are reported as odds ratios and 95% confidence intervals. Data were analysed using spss® 19.0 and stata® 12.^28^,^29^

**Table 1:** Risk for operative vaginal delivery and caesarean section in 185 primiparous women subjected to childhood sexual abuse (CSA) and 47 women subjected to rape in adulthood (RA), compared with 141 controls, from the general birth cohort

	Operative vaginal delivery OR (95% CI)				Caesarean section OR (95% CI)			
		
	Unadjusted	*P*	Adjusted	*P*	Unadjusted	*P*	Adjusted	*P*
Child sexual abuse (CSA)	0.80 (0.39–1.64)	0.54	0.84 (0.37–1.91)	0.60	1.65 (0.86–3.16)	0.13	1.41 (0.63–3.12)	0.40
Raped as adult (RA)	12.71 (5.09–31.73)	<0.01	12.17 (4.40–33.72)	<0.01	11.57 (4.48–29.41)	<0.01	9.92 (3.36–29.35)	<0.01
Younger (≤19 years of age)	0.34 (0.08–1.60)	0.18	0.38 (0.08–1.83)	0.23	0.19 (0.04–0.84)	0.03	0.34 (0.07–1.66)	0.18
Older (≥31 years of age)	2.57 (1.27–5.22)	0.01	2.55 (1.12–5.85)	0.03	2.40 (1.24–4.64)	0.01	1.65 (0.73–3.73)	0.23
Single (unsupported)	1.46 (0.66–3.22)	0.35			2.56 (1.20–5.46)	0.02		
Higher education (yes/no)	1.29 (0.67–2.48)	0.44	0.65 (0.30–1.43)	0.29	2.69 (1.49–4.85)	<0.01	2.12 (1.01–4.44)	0.05
Smoking (yes/no)	0.86 (0.42–1.73)	0.66			0.89 (0.48–1.66)	0.72		
Pregnancy termination and miscarriage	1.49 (0.77–2.90)	0.24			1.61 (0.89–2.90)	0.11		
Prenatal visits	0.98 (0.88–1.09)	0.73			1.07 (0.98–1.18)	0.14		
Height	0.99 (0.94–1.04)	0.71			1.00 (0.96–1.05)	0.93		
Pre-pregnant BMI (kg/m^2^)	1.00 (0.92–1.08)	0.95	0.96 (0.87–1.05)	0.36	1.12 (1.05–1.18)	<0.01	1.09 (1.02–1.17)	0.02
Epidural (yes/no)	2.63 (1.44–4.80)	<0.01	1.83 (0.92–3.64)	0.08	2.72 (1.58–4.68)	<0.01	2.89 (1.48–5.65)	0.01
Induced labour (yes/no)	1.41 (0.64–3.09)	0.39			3.29 (1.76–6.16)	<0.01		
Oxytocin augmentation (yes/no)	4.04 (1.89–8.62)	<0.01	2.43 (1.06–5.58)	0.04	1.01 (0.57–1.78)	0.97	0.43 (0.21.–0.90)	0.02
High obstetrical risk (high/low)	1.60 (0.81–3.16)	0.18	1.34 (0.64–2.82)	0.44	4.01 (2.18–7.35)	<0.01	3.07 (1.53–6.14)	<0.01
Blood loss > 500 ml	3.05 (1.39–6.70)	0.01			3.12 (1.54–6.31)	<0.01		
Apgar score <7 at 5 minutes	0.68 (0.08–6.08)	0.73			1.44 (0.36–5.87)	0.61		
Birthweight (hectogram)	1.09 (1.03–1.16)	0.01	1.07 (0.99–1.15)	0.08	1.08 (1.02–1.14)	0.01	1.09 (1.03–1.16)	0.01
Transferred to NICU (yes/no)	1.02 (0.33–3.12)	0.97	1.29 (0.37–4.45)	0.69	2.94 (1.34–6.46)	0.01	2.85 (1.09–7.52)	0.03

NICU, neonatal intensive care unit.

## Results

In the CSA group the average age at first childbirth was 24.3 years (range 14–37 years) versus 27.4 years (range 19–39 years) in the RA group and controls (*P* < 0.01). Sociodemographic characteristics, prenatal visits, obstetric risk and birth outcomes are presented in Table 2. There were significant differences between the groups in obstetric risks, induction/oxytocin augmentation, use of epidural analgesia and transferrals to neonatal intensive care (*P* < 0.01; Table 2). In the CSA group 18% were delivered by caesarean section, 9% by ventouse or forceps and 73% gave birth without operative interventions.

**Table 2:** Characteristics and birth outcomes of 373 primiparous women, 185 of whom had been subjected to childhood sexual abuse (CSA), 47 of whom had been raped in adulthood (RA) and 141 controls (CG), from the general birth cohort

Factor	CSA	RA	CG	χ^2^/*F*	*P*
	*n* = 185	*n* = 47	*n* = 141		
Single (unsupported) (%)	64 (35)	15 (32)	11 (8)	33.158	<0.01
Higher education (%)	59 (32)	21 (45)	75 (53)	15.163	<0.01
Not employed (%)	72 (39)	13 (28)	11 (8)	40.641	<0.01
Smokers (%)	70 (38)	17 (36)	28 (20)	12.848	<0.01
Height*	165 (145–180)	163 (149–176)	167 (150–185)	14.724	<0.01
Pre-pregnant BMI*	22.0 (15–41)	23.2 (17–45)	22.0 (17–37.5)	3.715	0.16
Pregnancy termination and miscarriage (%)	63 (34)	21 (45)	33 (23)	8.643	0.01
Prenatal visits*	11 (3–22)	12 (5–17)	12 (5–25)	0.587	0.75
Obstetric risk (%)	85 (46)	17 (36)	31 (22)	20.028	<0.01
Labour induced/augmented with oxytocin (%)	106 (57)	38 (81)	78 (55)	10.288	0.01
Epidural analgesia** (%)	76 (42)	26 (58)	43 (31)	11.137	<0.01
Apgar score <7 at 5 minutes (%)	9 (5)	1 (2)	2 (1)	3.257	0.20
Blood loss >500 ml (%)	30 (16)	8 (17)	19 (14)	0.590	0.74
Birthweight (*g*)*	3540 (700–4990)	3495 (896–4780)	3510 (2170–4700)	0.208	0.90
Transferred to neonatal intensive care unit (%)	24 (13)	9 (19)	6 (4)	10.836	<0.01
Mode of birth				59.350	<0.01
Vaginal without operative intervention (%)	135 (73)	10 (21)	108 (77)		
Ventouse or forceps (%)	17 (9)	20 (43)	17 (12)		
Caesarean section (%)	33 (18)	17 (36)	16 (11)		

* Median (range) Kruskall–Wallis test.

** Elective caesarean excluded.

**Figure 2:**
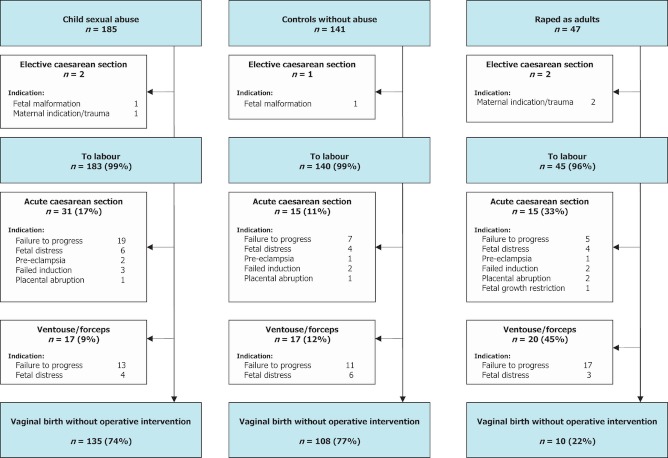
Flow chart for birth outcome and indications for operative delivery for 373 primiparous women: 185 with a history of child sexual abuse; 47 raped in adulthood; and 141 controls from the general birth cohort. Percentage of acute caesarean section, ventous and forceps deliveries and vaginal births, of the women going into labour.

In the RA group 36% were delivered by caesarean section, 43% by ventouse or forceps and 21% gave birth without operative interventions. In the control group 11% were delivered by caesarean section, 12% by ventouse or forceps and 77% gave birth without operative interventions (*P* < 0.01; Table 2). Figure 2 presents a flow chart for birth outcome and indications for operative deliveries. There were no significant differences between groups in indications for operative deliveries (*P* = 0.72).

Table 1 shows that the crude risks of operative vaginal delivery and caesarean section were 0.8 (95% CI 0.4–1.6) and 1.7 (95% CI 0.9–3.2), respectively, for the CSA group compared with controls. The crude risks for operative vaginal delivery and caesarean section for the RA group were 12.7 (95% CI 5.1–31.7) and 11.5 (95% CI 4.5–29.4), respectively, compared with controls (*P* < 0.01). The effects of the putative confounding factors on the relationship between abuse type and mode of delivery were generally marginal, and few of the adjustment effects seem noteworthy (Table 1). A slightly increased risk of operative vaginal delivery in the older group of women remains marginally significant, and augmentation with oxytocin is significant for caesarean section.

### Outcome of labour for vaginal birth

**Figure 3:**
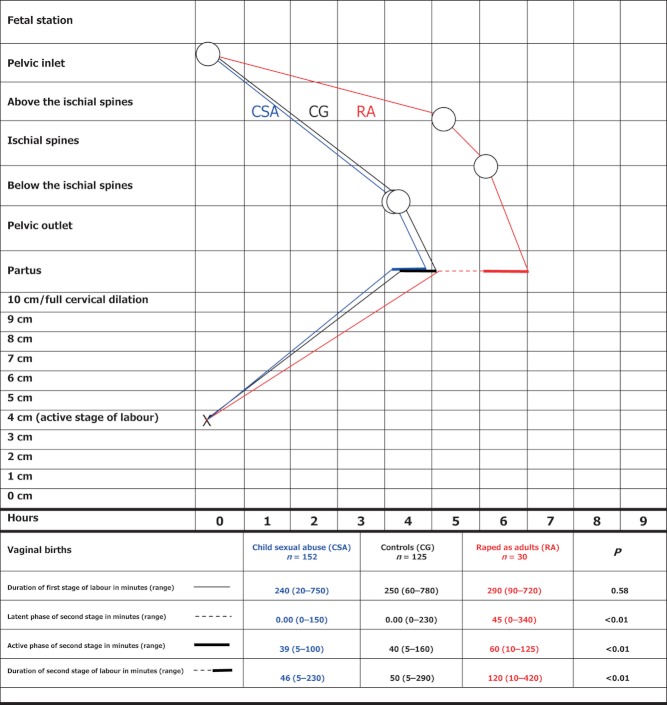
Schematic partogram: course, outcome and active labour in vaginal births in 307 primiparous women, 152 of whom were sexually abused as children (CSA), 30 of whom were raped as adults (RA) and 125 of whom were controls (CG). Median (range) Kruskall–Wallis test. 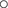, fetal part descending; 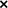, cervical dilation.

Figure 3 shows a schematic partogram in the three groups illustrated with median values in minutes. The duration of the second stage of labour was 46 minutes in the CSA group, 120 minutes in the RA group and 50 minutes in the control group (*P* < 0.01; Figure 3). Obstetric risk factors at the start of labour were 66 (43%) in the CSA group, 7 (23%) in the RA group and 25 (20%) in the control group (*P* < 0.01). Thirty-four women (22%) were induced in the CSA group, five women (17%) were induced in the RA group and 13 (10%) women were induced in the control group (*P* = 0.03). Seventy-six women (50%) were augmented with oxytocin in the CSA group, 24 women (80%) were augmented with oxytocin in the RA group and 68 women (54%) were augmented with oxytocin in the control group (*P* = 0.01). In the CSA group 57 women (35%) had an episiotomy versus 15 women (50%) in the RA group and 41 women (33%) in the control group (*P* = 0.20). There were 17 (11%) ventouse or forceps deliveries in the CSA group, 20 (67%) in the RA group and 17 (14%) in the control group (*P* < 0.001).

## Discussion

To our knowledge this is the first study comparing the labour outcomes in a group of nulliparous women who had been subjected to CSA with a group of nulliparous who had been subjected to RA. As compared with the control group from the general birth cohort at our hospital, women in the CSA group had no significant difference in rates of caesarean section and ventouse or forceps delivery, whereas women in the RA group had a 13-fold increase in risk for operative vaginal delivery and 12-fold increase in risk for caesarean section. Findings in the CSA group are in accordance with previous studies,^8^,^18^,^23^,^24^,^30^ but the differences between the CSA and RA groups have not previously been reported. The most common indications for operative vaginal delivery and acute caesarean sections in this study were failure to progress and fetal distress, which is in accordance with other studies.^31^

Surprisingly, all stages of labour in the CSA group were significantly shorter than in the RA group, and also somewhat shorter than the control group, and more women gave birth without operative interventions despite a higher proportion of women in this group having obstetric risk factors. The differences in duration of labour and birth outcomes in the CSA and RA groups are likely to be of complex origin. Age-related differences in physical and emotional maturity at the time of sexual abuse may influence later birthing strategies. Whereas children have to surrender to the perpetrator to a greater degree, and to withdraw emotionally from the traumatic experience, adults are able to face reality to a greater degree, and defend themselves by fight or flight and repression of the unavoidable traumatic experience.

Rhodes & Hutchinson have described four distinct birthing styles among women who have been subject to sexual abuse: fighting, taking control, surrendering and retreating.^32^ The ‘surrendering or retreating behaviour’ is recognisable among those in the CSA group, and may partly explain the shorter second stages seen in these women. During birth we have noticed that women with a history of CSA more often ‘give themselves over’ or withdraw mentally from the birth process, over which they have no control. Children subjected to abuse are powerless and often surrender without physically resisting the abuser.^32^,^33^ Another possible explanation may be that women subjected to abuse as children may have lost some of the natural resistance in the pelvic floor musculature. Many women with a history of CSA describe their lower abdomen and genitalia as being ‘without feeling’, ‘non-existent’ or constantly painful. Another factor that we also believe may be significant is the relationship to the abuser. Sexual access to a child is achieved more frequently by close, trusted people, in which trickery, coaxing or bribery are used as a means to commit sexual abuse, rather than the threats and violent force that often characterises rape in adulthood.

Women in the RA group had a second stage that was significantly longer than for women in the CSA group (120 versus 46 minutes), and it was the latent phase of the second stage in which this difference was most pronounced. They also had more ventouse or forceps deliveries. A rape in adulthood is often unexpected, and has a more violent character. It may seem as if the woman more or less unconsciously holds back, and resists letting the fetus descend through the pelvis and birth canal. We have previously described the reactivation of the rape trauma as a possible explanation for prolonged second stage.^25^ The labour strategies described by Rhodes & Hutchinson may explain part of the striking differences in the course and outcome of labour between the CSA and RA groups.

Another possible explanation for the high proportion of vaginal births in the CSA group may be their younger age.^27^ Also, the Norwegian Mother and Child cohort (MoBa) study, including data from 26 923 primiparas, showed that the number of instrumental vaginal births was lower among women reporting CSA than among women without such a report.^24^ The same study also showed a small but significant increase in caesarean section during labour in the group exposed to ‘any’ childhood abuse.^24^ In the studies by Leeners et al. and Benedict et al., CSA has not been associated with an increased risk for operative delivery.^8^,^18^ Our results for the CSA group are in accordance with these studies. Previous studies, however, have suggested an association between prolonged labour and sexual abuse in general.^8^,^25^,^32^ With no distinction between different forms and time points of the sexual abuse, possible differences between the CSA and RA groups may have disappeared in these studies.

Our study has limitations. There was little information on mental health status and abuse in our general birth cohort. Experiences of sexual abuse and assault are subjective, and cannot be assessed according to objective criteria. We have not been able to correct for emotional and personality-related factors, which may influence the way the woman herself interprets the experiences she has been subjected to. The women included had known psychosocial burdens and were referred to the mental health team at the University Hospital. This limits the generalisability of our findings with regard to labour outcomes in all women who have experienced sexual abuse or assault. The findings do indicate, however, that there may be differences in the duration of labour and outcome between different abuse groups. Future studies should concentrate on the reasons for these differences. Furthermore, it is important to determine what kind of help these women benefit from during pregnancy and childbirth.

## References

[1] Hulme PA (2000). Symptomatology and health care utilization of women primary care patients who experienced childhood sexual abuse. Child Abuse Negl.

[2] Chen LP, Murad MH, Paras ML, Colbenson KM, Sattler AL, Goranson EN (2010). Sexual abuse and lifetime diagnosis of psychiatric disorders: systematic review and meta-analysis. Mayo Clin Proc.

[3] Johnson CF (2004). Child sexual abuse. Lancet.

[4] Maniglio R (2009). The impact of child sexual abuse on health: a systematic review of reviews. Clin Psychol Rev.

[5] Hilden M, Schei B, Swahnberg K, Halmesmaki E, Langhoff-Roos J, Offerdal K (2004). A history of sexual abuse and health: a Nordic multicentre study. BJOG.

[6] Paras ML, Murad MH, Chen LP, Goranson EN, Sattler AL, Colbenson KM (2009). Sexual abuse and lifetime diagnosis of somatic disorders: a systematic review and meta-analysis. JAMA.

[7] Springer KW, Sheridan J, Kuo D, Carnes M (2003). The long-term health outcomes of childhood abuse An overview and a call to action. J Gen Intern Med.

[8] Leeners B, Richter-Appelt H, Imthurn B, Rath W (2006). Influence of childhood sexual abuse on pregnancy, delivery, and the early postpartum period in adult women. J Psychosom Res.

[9] Zinzow HM, Resnick HS, McCauley JL, Amstadter AB, Ruggiero KJ, Kilpatrick DG (2011). Prevalence and risk of psychiatric disorders as a function of variant rape histories: results from a national survey of women. Soc Psychiatry Psychiatr Epidemiol.

[10] Ellsberg M, Jansen HA, Heise L, Watts CH, Garcia-Moreno C (2008). Intimate partner violence and women's physical and mental health in the WHO multi-country study on women's health and domestic violence: an observational study. Lancet.

[11] Faravelli C, Giugni A, Salvatori S, Ricca V (2004). Psychopathology after rape. Am J Psychiatry.

[12] Eberhard-Gran M, Schei B, Eskild A (2007). Somatic symptoms and diseases are more common in women exposed to violence. J Gen Intern Med.

[13] Wijma B, Schei B, Swahnberg K, Hilden M, Offerdal K, Pikarinen U (2003). Emotional, physical, and sexual abuse in patients visiting gynaecology clinics: a Nordic cross-sectional study. Lancet.

[14] Campbell R, Wasco SM (2005). Understanding rape and sexual assault: 20 years of progress and future directions. J Interpers Violence.

[15] Pereda N, Guilera G, Forns M, Gomez-Benito J (2009). The prevalence of child sexual abuse in community and student samples: a meta-analysis. Clin Psychol Rev.

[16] Leeners B, Stiller R, Block E, Gorres G, Rath W (2010). Pregnancy complications in women with childhood sexual abuse experiences. J Psychosom Res.

[17] Stoltenborgh M, van Ijzendoorn MH, Euser EM, Bakermans-Kranenburg MJ (2011). A global perspective on child sexual abuse: meta-analysis of prevalence around the world. Child Maltreat.

[18] Benedict MI, Paine LL, Paine LA, Brandt D, Stallings R (1999). The association of childhood sexual abuse with depressive symptoms during pregnancy, and selected pregnancy outcomes. Child Abuse Negl.

[19] Johnson JK, Haider F, Ellis K, Hay DM, Lindow SW (2003). The prevalence of domestic violence in pregnant women. BJOG.

[20] Nerum H, Halvorsen L, Sorlie T, Oian P (2006). Maternal request for cesarean section due to fear of birth: can it be changed through crisis-oriented counseling?. Birth.

[21] Lukasse M, Vangen S, Oian P, Kumle M, Ryding EL, Schei B (2010). Childhood abuse and fear of childbirth–a population-based study. Birth.

[22] Lukasse M, Schei B, Vangen S, Oian P (2009). Childhood abuse and common complaints in pregnancy. Birth.

[23] Van der Hulst LAM, Bonsel GJ, Eskes M, Birnie E, Van Teijlingen E, Bleker OP (2006). Bad experience, good birthing: Dutch low-risk pregnant women with a history of sexual abuse. J Psychosom Obstet Gynaecol.

[24] Lukasse M, Vangen S, Oian P, Schei B (2010). Childhood abuse and caesarean section among primiparous women in the Norwegian Mother and Child Cohort Study. BJOG.

[25] Nerum H, Halvorsen L, Oian P, Sorlie T, Straume B, Blix E (2010). Birth outcomes in primiparous women who were raped as adults: a matched controlled study. BJOG.

[26] Halvorsen L, Nerum H, Sorlie T, Oian P (2010). Does counsellor's attitude influence change in a request for a caesarean in women with fear of birth?. Midwifery.

[27] Noll JG, Shenk CE, Putnam KT (2009). Childhood sexual abuse and adolescent pregnancy: a meta-analytic update. J Pediatr Psychol.

[28] Statistics P SPSS 19. SPSS Inc Chicago.

[29] Software SS, StataCorp (2011). STATA 12. Lakeway Drive.

[30] Tiwari A, Chan KL, Fong D, Leung WC, Brownridge DA, Lam H (2008). The impact of psychological abuse by an intimate partner on the mental health of pregnant women. BJOG.

[31] Kolas T, Hofoss D, Daltveit AK, Nilsen ST, Henriksen T, Hager R (2003). Indications for cesarean deliveries in Norway. Am J Obstet Gynecol.

[32] Rhodes N, Hutchinson S (1994). Labor experiences of childhood sexual abuse survivors. Birth.

[33] Parratt J (1994). The experience of childbirth for survivors of incest. Midwifery.

